# Prediction of disease-related metabolites using bi-random walks

**DOI:** 10.1371/journal.pone.0225380

**Published:** 2019-11-15

**Authors:** Xiujuan Lei, Jiaojiao Tie

**Affiliations:** School of Computer Science, Shaanxi Normal University, Xi’an China; University of York, UNITED KINGDOM

## Abstract

Metabolites play a significant role in various complex human disease. The exploration of the relationship between metabolites and diseases can help us to better understand the underlying pathogenesis. Several network-based methods have been used to predict the association between metabolite and disease. However, some methods ignored hierarchical differences in disease network and failed to work in the absence of known metabolite-disease associations. This paper presents a bi-random walks based method for disease-related metabolites prediction, called MDBIRW. First of all, we reconstruct the disease similarity network and metabolite functional similarity network by integrating Gaussian Interaction Profile (GIP) kernel similarity of diseases and GIP kernel similarity of metabolites, respectively. Then, the bi-random walks algorithm is executed on the reconstructed disease similarity network and metabolite functional similarity network to predict potential disease-metabolite associations. At last, MDBIRW achieves reliable performance in leave-one-out cross validation (AUC of 0.910) and 5-fold cross validation (AUC of 0.924). The experimental results show that our method outperforms other existing methods for predicting disease-related metabolites.

## Introduction

Metabolites play an important role in the maintenance, growth and reproduction of organisms, and are greatly helpful to illustrate the underlying molecular disease-causing mechanisms [1]. There is abundant evidence that diseases are always accompanied with changes in metabolite [2]. Hence, it is significant to identify abnormal metabolites for diagnosis and treatment of diseases [3].

As the development of molecular technology, many researchers have revealed the association between disease and other molecular products like gene, microRNA, circRNA, protein, etc [4–6]. Luo et al. used BIRW to predict the potential association between drug and disease [7]. Yan et al. developed the method DNRLMF-MDA by integrating disease similarity and miRNA similarity to predict disease-related miRNA based on dynamic neighbourhood regularized logistic matrix factorization [8]. In recent years, more and more researchers have been attracted to metabolite. Czech et al. used the method of gas and liquid chromatography-tandem mass spectrometry (GC-MS and LC-MS/MS) to analyze CSF samples in Alzheimer's patients [9]. An integrated mass spectrometry approach was developed to research the new cerebrospinal fluid biomarkers of multiple sclerosis [10]. The contents of metabolites in the patients of Alzheimer's brain were studied in [11]. In 2010, Erika et al. developed a method to discover phenylbutyrate metabolites in patients with Huntington’s disease [12]. Susan et al. integrated metabolomics and transcriptomes data to identify biomarkers for type 2 diabetes [13]. Baumgartner et al. proposed a novel network-based approach to identifying dynamic metabolic biomarkers in cardiovascular disease [14]. Previous research has shown that metabolites with similar functions are highly likely to be associated with the same or similar diseases [3]. Shang et al. proposed a method named PROFANCY to predict metabolites associated with disease based on metabolite functional similarity in metabolic pathways [15]. Hu et al. constructed a weighted metabolite association network for all the similarities of metabolite pairs, the random walk was utilized to predict metabolic markers of diseases [16]. Although some achievements has been made, there are still a lot of researches to do in the field of disease-related metabolites prediction. Considering that traditional RWR cannot fully combine the information of the metabolite network, disease network and disease-metabolite association network, and cannot predict the disease-related metabolites without known relationships. We apply bi-random walks algorithm to predict metabolite-disease associations by walking in disease network and metabolite network.

In this study, we utilize bi-random walks to identify disease-related metabolites. First, we compute disease semantic similarity and metabolite functional similarity, as well as create the Gaussian Interaction Profile kernel similarity for diseases and metabolites base on known metabolite-disease associations. Then, we integrate disease semantic similarity and disease GIP kernel similarity to construct disease similarity network. Similarly, metabolite functional similarity and metabolite GIP kernel similarity are integrated to construct metabolite similarity network. After that, Bi-random walks is used in two subnetworks to predict metabolite-disease associations. Finally, leave-one out cross validation, five-fold cross validation and case studies are used to assess the performance of our method. The experimental results illustrate that our method MDBIRW can effectively predict disease-related metabolites and show the superior performance compared to other competing methods.

## Materials and methods

### Human metabolite-disease association

We downloaded the metabolites data and diseases data from Human Metabolome Database (HMDB) [17] and Human Disease Ontology (DO) [18], respectively (S1 File). 2262 metabolites, 216 diseases and 4537 metabolite-disease associations can be obtained after removing redundant associations. The set of metabolites are denoted by $M={\left\{m_{i}\right\}}_{i=1}^{m}$, where *m* is the number of metabolites. Similarly, the set of diseases is denoted by $D={\left\{d_{j}\right\}}_{j=1}^{n}$, where *n* is the number of diseases. And the adjacent matrix A indicates the metabolite-disease associations network. If there is a known association between disease *d*(*i*) and metabolite *m*(*j*), *A* (*i*, *j*) is equal to 1, otherwise 0.

### Disease semantic similarity

In the MeSH database(https://meshb.nlm.nih.gov/) [19], every disease can be regarded as a node in Directed Acyclic Graph (DAG). Each MeSH descriptor displays a hierarchical DAG structure. For disease *d* which can be represented as DAG(*d*) = (*d*, T(*d*), E(*d*)), where T(*d*) is an ancestral set of disease *d*_*i*_ and E(*d*) indicates the corresponding edges. The semantic score of disease *d* can be calculated as follows:

$$D_{d}(t)=\left\{ \begin{array}{l} \;1,\;if\;t=d\\ max\{\Delta *D_{d}(t^{\prime })|t^{\prime }\in children\;of\;t\},\;if\;t\neq d \end{array}\right.$$ (1)

where the disease *t*∈*T*(*d*), Δ is semantic contribution decay factor and we set Δ = 0.5.

The semantic value DV(*d*) of disease *d* is defined as follows:

$$\mathrm{DV}(d)=\Sigma_{t\in T(d)}D_{d}(t)$$ (2)

Then, the semantic similarity between *d*_*i*_ and *d*_*j*_ can be calculated as follows:

$$S\;\left(d_{i},\;d_{j}\right)=\frac{\sum_{t\in T\left(d_{i}\right)\cap T\left(d_{j}\right)}\left(D_{d_{i}}(t)+D_{d_{j}}(t)\right)}{DV\left(d_{i}\right)+DV\left(d_{j}\right)}$$ (3)

where DV (*d*_*i*_) and DV (*d*_*j*_) indicate the value of the disease t associated with disease *d*_*i*_ and *d*_*j*_. Finally, we obtain the disease semantic similarity among all diseases, and symmetric matrix $S_{n\times n}^{d}$ indicates the disease semantic similarity network.

### Metabolite functional similarity

Wang et al. proposed a method called MISIM [20]. In previous work, MISIM was used to calculate the similarity of micro-RNAs based on the similarity of related diseases. We apply the MISIM to compute the similarity of metabolites by using the related diseases semantic similarity. Here, we define *d* as a specific disease and *D* = {*d*_1_,*d*_2_,⋯,*d*_*k*_} represent a disease group. The similarity of disease *d* to group of diseases *D* can be calculated as follows:

$$S\;(d,D)=\max_{1\leq i\leq k}\left(d,d_{i}\right)$$ (4)

where *k* represents the number of *D*, S (*d*, *D*) represents the maximum similarity between one disease and a group of diseases.

Afterwards, we can obtain the similarity of metabolites by the following formula:

$$S_{m}(m_{i},m_{j})=\frac{\sum_{1\leq k\leq {num}_{i}}S(d_{ik},D_{j})+\sum_{1\leq k\leq {num}_{j}}S(d_{jk},D_{i})}{{num}_{i}+{num}_{j}}$$ (5)

where D_*i*_ and D_*j*_ are two sets of diseases related to metabolite *m*_*i*_ and m_*j*_, *num*_*i*_ and *num*_*j*_ represent the number of *D*_*i*_ and *D*_*j*_, respectively. The symmetric matrix $S_{m\times m}^{m}$ indicates the metabolic functional similarity network.

### Gaussian interaction profile kernel similarity for diseases and metabolites

Considering the assumption that the more common metabolites(diseases) of a disease(metabolite) pair has, the more similar they are. We utilize Gaussian Interaction Profile kernel similarity to calculate metabolite similarity and disease similarity based on the topologic information of known disease-metabolite associations.

In the disease-metabolite association network *A*, IP (*m*_*i*_) represents the interaction profile for metabolite *m*_*i*_, which is a binary vector with size of *n*. If a disease is related to *m*_*i*_, the corresponding value of IP (*m*_*i*_) is 1, otherwise 0. According to the interaction profiles, the Gaussian interaction profile kernel similarity matrix for metabolite *GS*_*m*_ can be calculated as follows:

$${GS}_{m}(i,j)=\exp\left(-\lambda_{d}{\left\|IP(m_{i})-IP(m_{j})\right\|}^{2}\right)$$ (6)

$$\lambda_{d}=\lambda_{d}^{\prime }/(\frac{1}{m}\sum_{i=1}^{m}{\left\|IP(m_{i})\right\|}^{2})$$ (7)

where *m* is the number of metabolites. *λ*_*d*_ indicates the normalized kernel bandwidth, and can be updated by a new normalized bandwidth $\lambda_{d}^{\prime }$. According to previous relevant research, we set $\lambda_{d}^{\prime }=1$ [21].

Similarly, we can compute the Gaussian interaction profile kernel similarity matrix for diseases *GS*_*d*_ as follows:

$${GS}_{d}(i,j)=exp(-\lambda_{m}{\left\|IP(d_{i})-IP(d_{j})\right\|}^{2})$$ (8)

$$\lambda_{m}=\lambda_{m}^{\prime }/(\frac{1}{n}\sum_{i=1}^{n}{\left\|IP(d_{i})\right\|}^{2})$$ (9)

where $\lambda_{m}^{\prime }$ is also set as 1, *n* is the number of diseases.

### Reconstruction of disease similarity network and metabolite similarity network

In this section, we reconstruct disease similarity and metabolite similarity. A disease similarity network can be reconstructed based on the disease semantic similarities and gaussian interaction profile kernel similarity of disease. We define the disease similarity network DS on the basis of matrix S^d^ and *GS*_*d*_ as follows:

$$DS(i,j)=\left\{ \begin{array}{l} \;{GS}_{d}(i,j),\;if\;S^{d}(i,j)=0\\ \frac{S^{d}(i,j)+{GS}_{d}(i,j)}{2},\;if\;S^{d}(i,j)\neq 0 \end{array}\right.$$ (10)

where *DS*(*i*,*j*) is the final disease similarity value of disease *i* and disease *j*. When the disease semantic similarity *S*^*d*^(*i*,*j*) = 0, we replace *S*^*d*^(*i*,*j*) with *GS*_*d*_(*i*,*j*). Otherwise, we hypothesize that the disease semantic similarity is as important as the Gaussian Interaction Profile Kernel Similarity of disease.

Similarly, metabolite similarity network MS can be reconstructed by *S*^*m*^ and *GS*_*m*_, the final metabolite similarity network can be calculated as follows:

$$MS(i,j)=\left\{ \begin{array}{l} \;{GS}_{m}(i,j),\;if\;S^{m}(i,j)=0\\ \frac{S^{m}(i,j)+{GS}_{m}(i,j)}{2},\;if\;S^{m}(i,j)\neq 0 \end{array}\right.$$ (11)

where *MS*(*i*,*j*) represents the similarity value between metabolite *i* and metabolite *j*.

### Bi-Random walks on heterogeneous network

**Fig 1:**
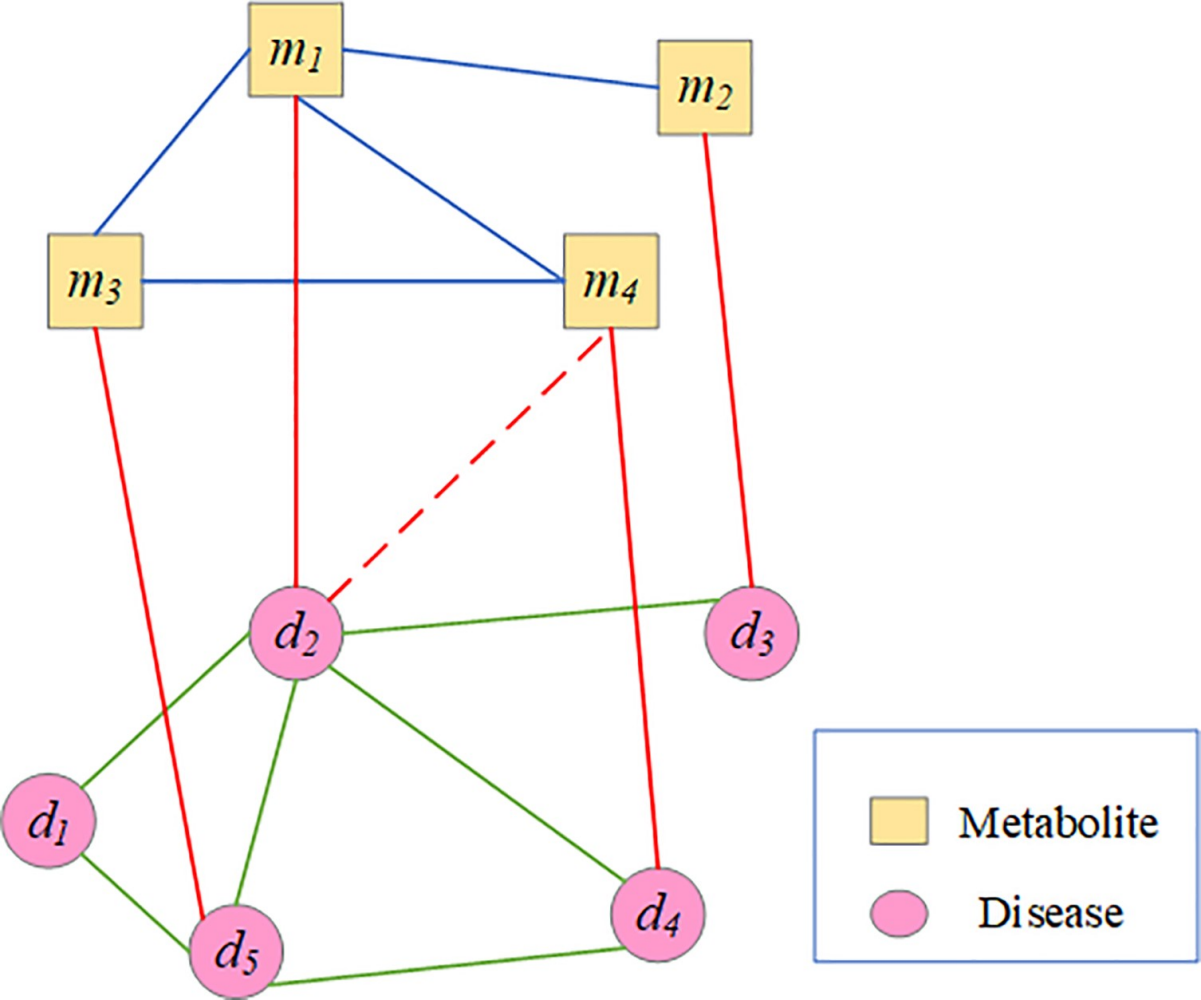
An example of the heterogeneous network. The blue edges indicate the metabolite similarity between metabolites, green edges indicate the disease similarity between diseases, and red edges between diseases and metabolites, which indicate the known metabolite-disease associations, and the dashed edges between metabolites and diseases indicate the novel association.

**Fig 2:**
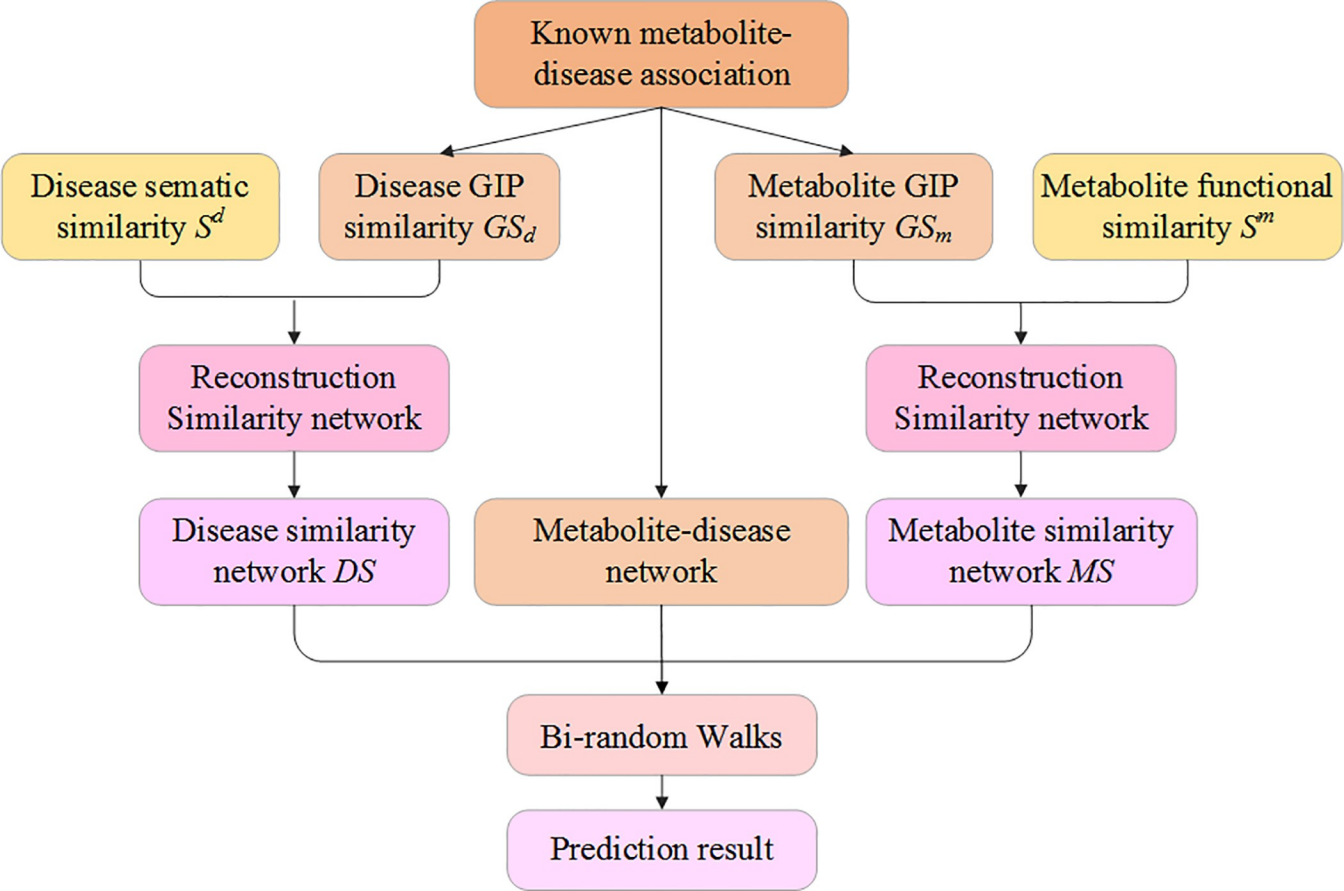
The workflow of MDBIRW for predicting disease-related metabolite.

In this study, we propose a novel method to predict metabolite-disease associations. With disease similarity network, metabolite similarity network and known disease-metabolite network, we create a Heterogeneous network, including two types of nodes and three types of edges among them. Fig 1 is an example of the heterogeneous network. The upper sub-network is a metabolite similarity network, and the lower sub-network is a disease similarity network. The middle sub-network is a bipartite graph of metabolite-disease relationship. Supposing *m*_*1*_ and *m*_*4*_ have a high similarity value, and *m*_*1*_ has a known association with *d*_*2*_. In order to predict the association between *m*_*4*_ and *d*_*2*_, we can take *m*_*4*_ as starting node for random walker, which jump from *m*_*4*_ to *m*_*1*_ and then to *d*_*2*_ through the edge that connect to *m*_*1*_ and *d*_*2*_. we also can take *d*_*2*_ as starting node for random walker, which jump from *d*_*2*_ to *d*_*4*_ and the to *m*_*4*_. These two ways both can obtain the associated probability between *m*_*4*_ and *d*_*2*_, the former firstly finds out the most similar intermediate metabolites based on the similarity of metabolites, and then calculates the associated probability between intermediate metabolites and corresponding diseases based on intermediate metabolites. Using bi-random walks algorithm [22, 23] can achieve forecast by walking in metabolite subnetwork and disease subnetwork. The associated probability of arbitrarily metabolite-disease can be calculate by bi-random walk. Fig 2 shows the workflow of MDBIRW for predicting disease-related metabolite.

Bi-random walks is utilized to evaluate potential metabolite-disease association, the association probability of metabolite-disease pair without known association record would be computed based on the steady state of the random walk process. During the random walk, metabolite subnetwork and disease subnetwork have different walking steps. Different walking steps can better obtain information of direct or undirect nodes in different networks. Hence, we define *l*, *r* as the numbers of maximal iterations in the metabolite subnetwork and disease subnetwork. The process of bi-random walks is described as follows:

$${RM}^{t}=\alpha \;MS∙{RM}^{t-1}+(1-\alpha )A$$ (12)

$${RD}^{t}=\alpha \;{RD}^{t-1}∙DS+(1-\alpha )A$$ (13)

Here, α represents the decay factor with ranges from 0 to 1. *RM*^*t*^(*i*,*j*) and *RD*^*t*^(*i*,*j*) denote the probability of walking on metabolite similarity network and disease similarity network, respectively. MDBIRW can eliminate bias caused by topological and structural characteristics of the different networks by adjusting the number of walking steps of metabolite subnetwork and disease subnetwork. The pseudocode of MDBIRW algorithm is shown in Algorithm 1.

**Algorithm 1. Algorithm for predicting the potential associations between metabolites and diseases**

**Input:** Disease set *D*, metabolite set *M* and metabolite-disease adjacency matrix *A*, parameter α, *l* and *r*

**Output:** predicted association matrix *R*

1: Calculate disease semantic similarity *S*^*d*^ and metabolite functional similarity *S*^*m*^;

2: Calculate disease GIP kernel similarity *GS*_*d*_ and metabolite GIP kernel similarity *GS*_*m*_;

3: Construct the disease similarity matrix *DS* and metabolite similarity matrix *MS*;

4: Normalize DS and MS to *DS*^***’***^ and *MS*^***’***^, respectively;

5: *R*_*0*_ = *A* = *A* / sum(*A*);

6: for t = 0 to max (*l*, *r*);

7:    flagm = flagd = 1;

8:    if t < = *l*

9:     *RM* = α MS′∙*RM*^*t*−1^+(1−α)A;

10:     flagm = 1;

11:    end if

12:    if t < = *r*

13:        *RD* = α *RD*^*t*−1^∙DS′+(1−α)A;

14:      flagd = 1;

15:    end if

16:    *R* = (flagm * *RM* + flagd * *RD*) / (flagm + flagd);

17: end for

18: **return** *R*

## Results

### Parameter analysis

**Fig 3:**
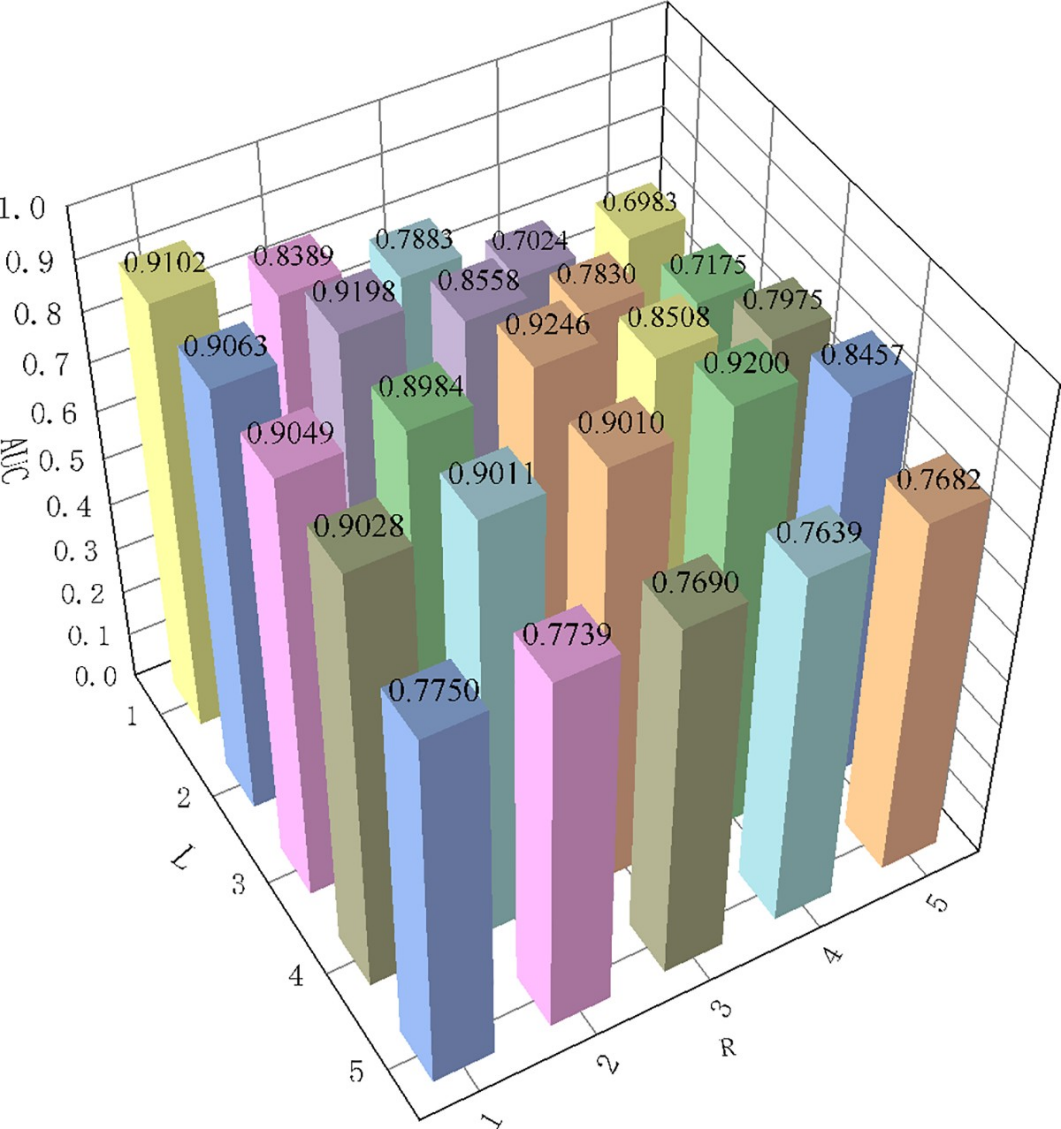
Description of transition probability α = 0.3. Fix the α is 0.3, values of *l* and *r* is {1,2,3,4,5}. When *l* and *r* is equal to 3, obtain the maximum AUC value of 0.924.

Three parameters *l*, *r*, and *α* are probed in MDBIRW. The parameter *α* is decay factor, the range of *α* is {0.3,0.5,0.7,0.9}. *l* and *r* control the iteration steps of two subnetwork, and choose the two parameters from {1,2,3,4,5}. If *l* > *r* means the random walker prefer to walk in metabolite network, vice versa. The analysis results of parameters are shown as Table 1 and the bar chart of α = 0.3 is shown in Fig 3.

**Table 1:** The analysis results of parameters.

α = 0.3		*r* = 1	*r* = 2	*r* = 3	*r* = 4	*r* = 5
	*l* = 1	0.910	0.838	0.788	0.702	0.698
	*l* = 2	0.906	0.919	0.855	0.782	0.717
	*l* = 3	0.904	0.898	0.924	0.850	0.797
	*l* = 4	0.902	0.901	0.900	0.920	0.845
	*l* = 5	0.774	0.773	0.768	0.763	0.768
α = 0.5		*r* = 1	*r* = 2	*r* = 3	*r* = 4	*r* = 5
	*l* = 1	0.911	0.861	0.838	0.778	0.719
	*l* = 2	0.905	0.911	0.859	0.828	0.776
	*l* = 3	0.896	0.898	0.909	0.859	0.819
	*l* = 4	0.899	0.894	0.895	0.907	0.842
	*l* = 5	0.786	0.775	0.768	0.765	0.761
α = 0.7		*r* = 1	*r* = 2	*r* = 3	*r* = 4	*r* = 5
	*l* = 1	0.917	0.877	0.836	0.818	0.785
	*l* = 2	0.889	0.906	0.857	0.833	0.806
	*l* = 3	0.877	0.887	0.904	0.859	0.827
	*l* = 4	0.871	0.867	0.873	0.904	0.847
	*l* = 5	0.784	0.774	0.769	0.765	0.766
α = 0.9		*r* = 1	*r* = 2	*r* = 3	*r* = 4	*r* = 5
	*l* = 1	0.917	0.867	0.840	0.835	0.808
	*l* = 2	0.883	0.895	0.863	0.834	0.818
	*l* = 3	0.851	0.858	0.892	0.834	0.819
	*l* = 4	0.844	0.834	0.832	0.878	0.829
	*l* = 5	0.778	0.776	0.776	0.772	0.759

The range of α is {0.3,0.5,0.7,0.9}. The range of l and r is {1,2,3,4,5}.

We explore the influences of *l* and *r* by using grid search method. From Table 1, we can conclusion that the maximum iteration steps *l* and *r* should not exceed 4. The AUC values on the diagonal is almost always higher than the rest values of its row and column. In other words, the optimal AUC value will be obtained when the maximum iteration steps of metabolite network and disease network are equal. Therefore, in our study, the optimal parameters are set that *α* = 0.3,*l* = 3,*r* = 3.

### Performance of MDBIRW

Leave one out cross-validation (LOOCV) only take one sample as test set and the remains are used as training data. In our study, there are 2262 metabolites, coupled with 216 diseases and 4537 metabolite-disease associations. Therefore, we need to execute LOOCV program 4537 times. At each round, one corresponding known metabolite-disease association should be converted to unknown as test sample and the rest of known metabolite-disease association be used to as training samples. After execute bi-random walks with LOOCV, predicted results will be obtained.

Five-fold cross-validation (FFCV) is also utilized to evaluate the performance of our method. In FFCV, 4537 metabolite-disease associations were randomly divided into 5 groups. For each execution, one group is used as test set while 4 groups are used as training sets [24].

Receiver Operating Characteristic (ROC) curve is also called sensitivity curve, which using false positive rate and true positive rate as horizontal axis and vertical axis, respectively. The area of under the ROC curve is AUC value. The higher AUC value is, the better performance will be. In our study, the number of negative samples is more than the number of positive samples. Hence, we randomly select as many negative samples as positive samples. We arrange the final predicted values in descending order and calculate false positive rate and true positive rate by setting thresholds. Finally, the true positive rate (*TPR*) and false positive rate (*FPR*) for each threshold can be computed as follows:

$$TPR=\frac{TP}{TP+FN}$$ (14)

$$FPR=\frac{FP}{FP+TN}$$ (15)

where *TP* and *TN* represent the number of positive samples and negative samples that can be correctly identified, and *FP* and *FN* are the number of the positive samples and negative samples that cannot be correctly identified, respectively.

Precision-Recall (PR) curve utilizes recall and precision as horizontal axis and vertical axis of PR curve. The area under precision-recall curve (AUPR) is to evaluate the performance of our method by considering the precision and recall. Different precision-recall pairs will be obtained by setting different thresholds. Precision and recall can be calculated as follows:

$$Precision=\frac{TP}{TP+FP}$$ (16)

$$Recall=\frac{TP}{TP+FN}$$ (17)

where *TP* indicates the number of real identified positive samples. *FP* and *FN* respectively represent the number of negative samples that are incorrectly labelled as positive samples and the number of positive samples that are incorrectly labelled as negative samples.

**Fig 4:**
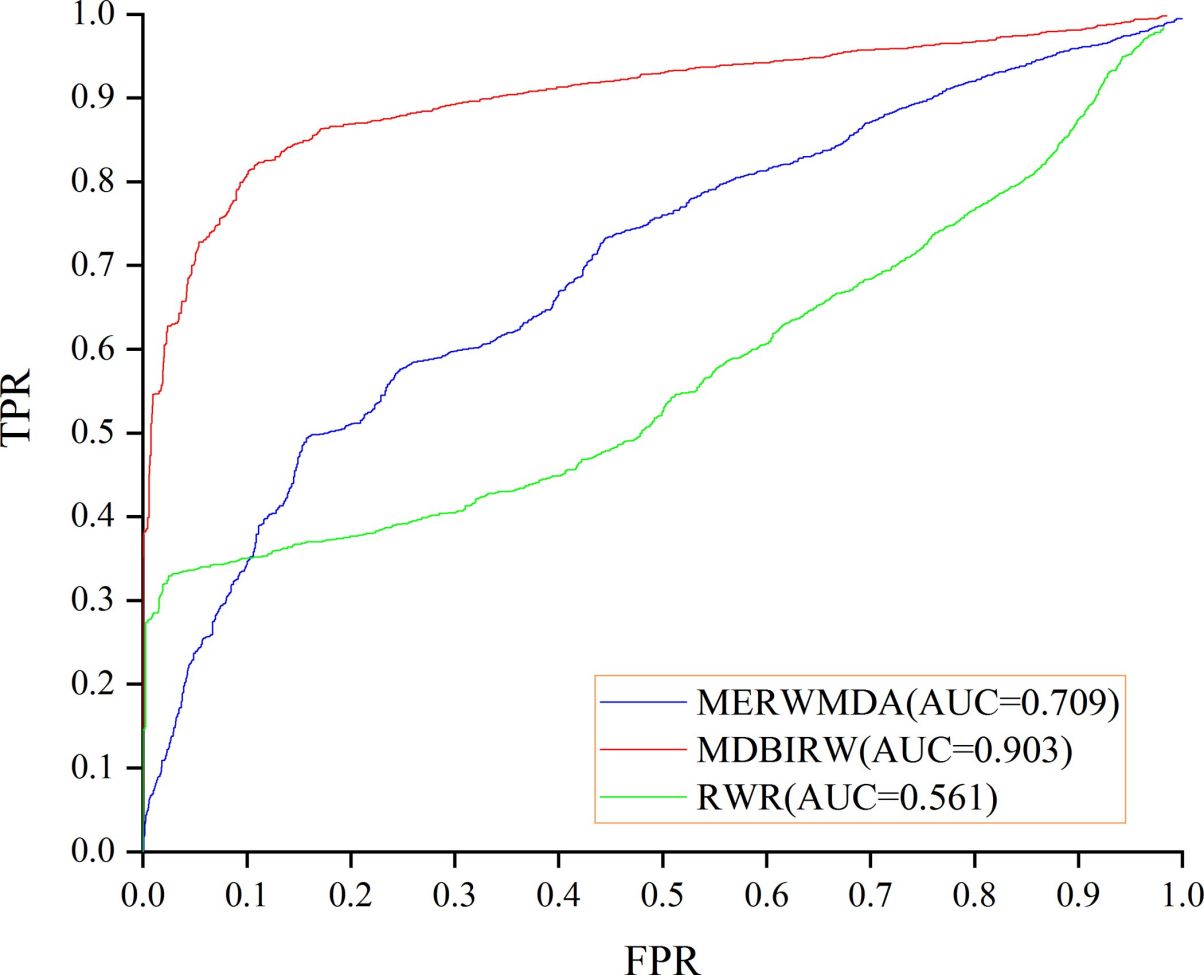
The comparison result of MDBIRW, MERWMDA and RWR in LOOCV.

**Fig 5:**
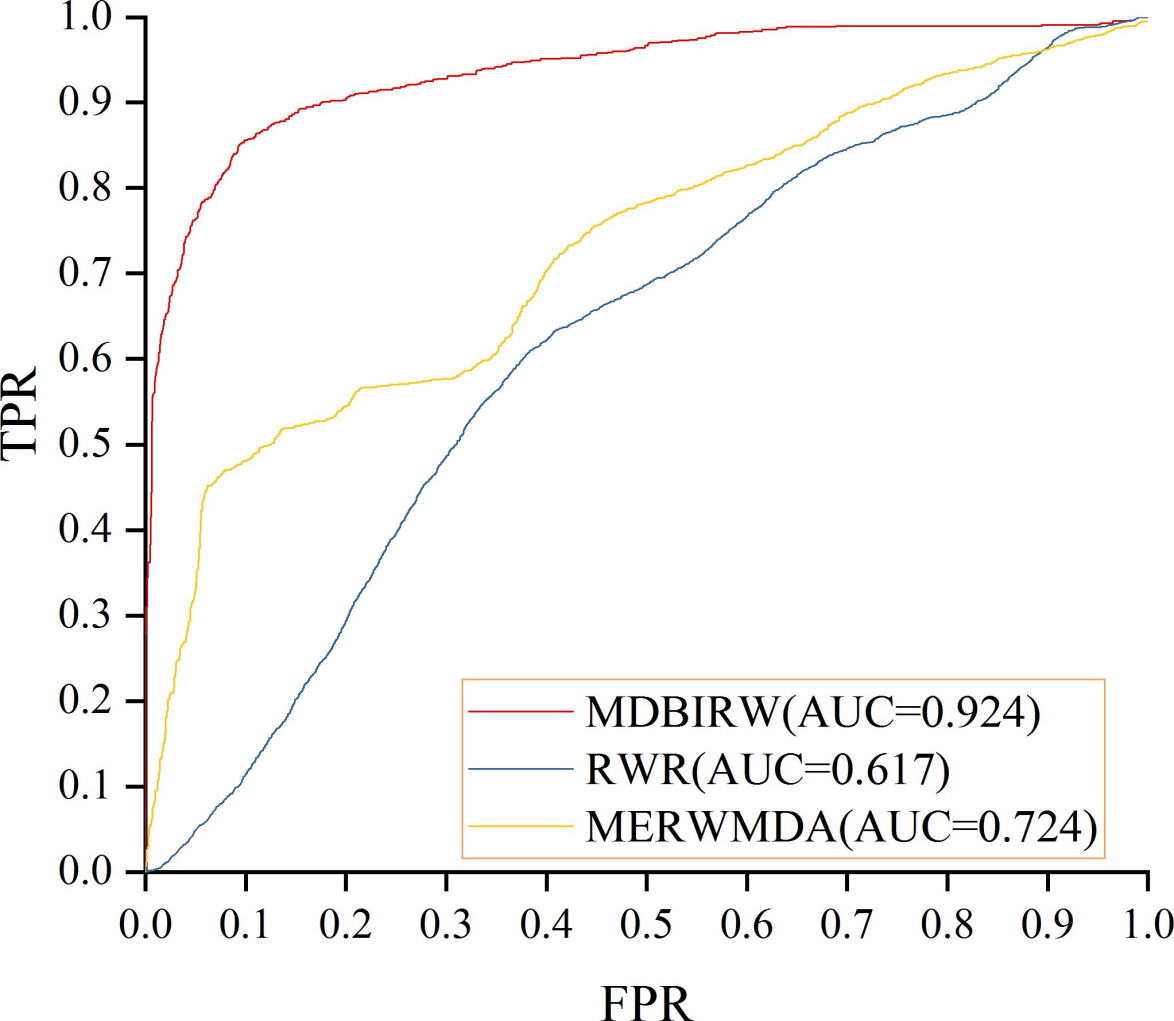
The comparison result of MDBIRW, MERWMDA and RWR in FFCV.

**Fig 6:**
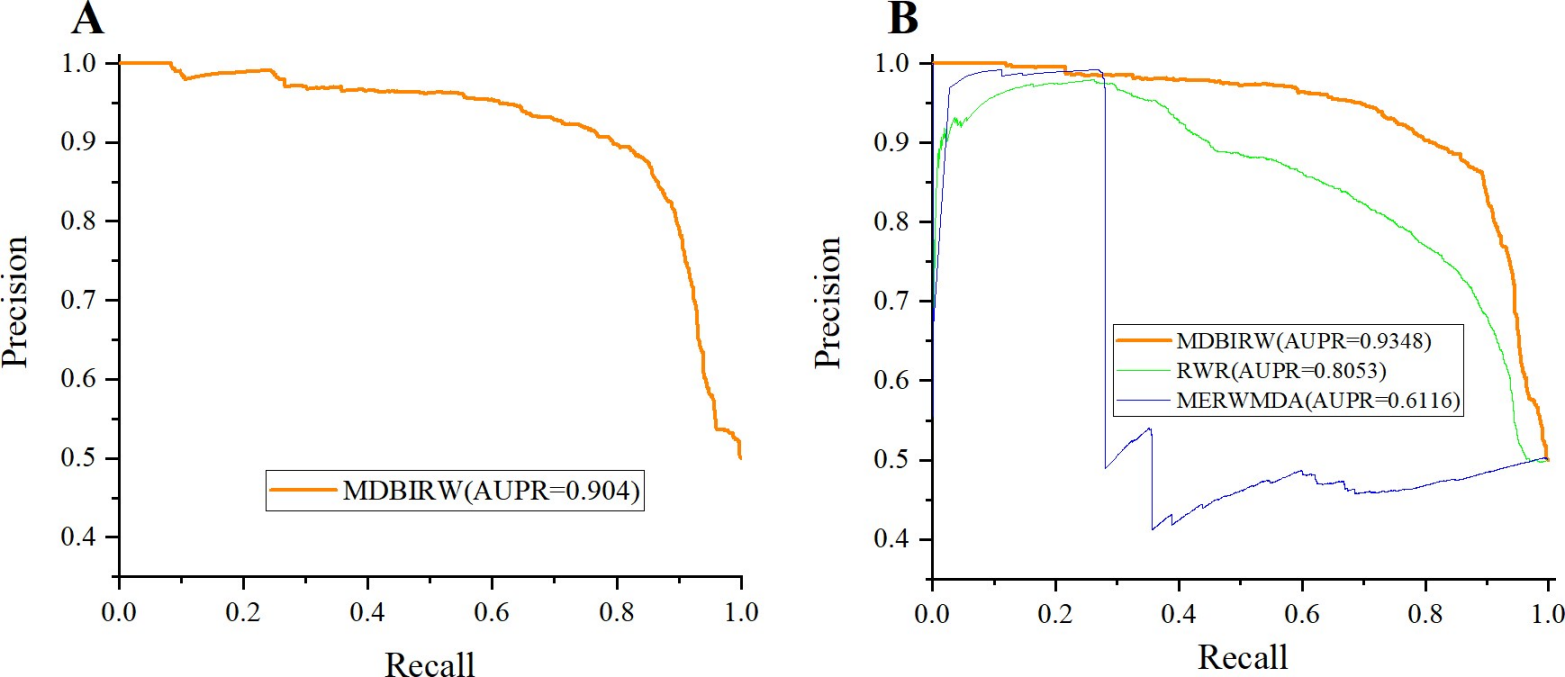
The PR curve of MDBIRW. (A) PR curve in LOOCV with AUPR = 0.904. (B)PR curves of MDBIRW, MERWMDA and RWR in FFCV.

According to our predicted result, the result of LOOCV is 0.903 and FFCV is 0.924, which confirms the superior performance of our method. Fig 4 shows the comparison result of LOOCV. MERWMDA [25] applied the maximum entropy theory to the random walk and revealed potential disease-miRNA associations on the heterogeneous network. RWR [26], the traditional random walk with restart algorithm, starting from any node and it have two choices in each step: randomly moving to neighbor nodes with (1−α) or returning to start node with probability α. MERWMDA and RWR are utilized as comparison methods to verify the performance of our method. Fig 5 shows the comparison result of MDBIRW, MERWMDA and RWR in FFCV. ROC and PR curves are plotted to evaluate the performance of our method. We use same number of positive and negative samples, the trend of these two curves is similar. Fig 6 shows PR curve of MDBIRW in LOOCV and FFCV.

### Case studies

We chose obesity, colorectal cancer and Alzheimer's disease as case studies. For each disease, we removed all known metabolite-disease associations and performed MDBIRW to obtain predicted scores. According the experimental prediction score (from high to low), we obtain the top 10 metabolites related to disease. Next, we mined biomedical literature from the National Center for Biotechnology information (NCBI, https://www.ncbi.nlm.nih.gov/) database and manually checked these metabolites. As a result, 8 out of 10, 9 out of 10 and 10 out of 10 predicted obesity, colorectal cancer and Alzheimer's disease be validated, respectively.

Obesity has become a major public health problem around the world, the prevalence rate of which is rising in almost all countries. There is growing evidence that obesity is linked to metabolite. As shown in Table 2, by executing bi-random walks to identify underlying metabolites with obesity, nine of top 10 identified metabolites have been validated. The change of L-Phenylalanine in obese men suggested the early changes in obesity in young men [27].The levels of Cholesterol is relatively high in obese young men has been already verified [27]. Zhao et al. has discovered that the levels of glycine have significant weight at baseline during five years [28]. L-Tryptophan and L-Tyrosine are abnormally expressed in obese children [28]. The changes of L-Arginine and L-Histidine were positively with obese parameter [29]. Central adiposity is associated with creatine changes, which has been found by Kaur et al [30]. 5-Hydroxyindole acetic acid and L-Alanine have not been confirmed link to obesity in human.

**Table 2:** Top 10 metabolites of obesity identified by bi-random walks method.

Metabolite Names	HMDB ID	Evidences
L-Phenylalanine	HMDB0000159	PMID: 21890434
Cholesterol	HMDB0000067	PMID: 25725317
Glycine	HMDB0000123	PMID: 27708848
L-Tryptophan	HMDB0000929	Simone et al.,2013
L-Histidine	HMDB0000177	PMID:25700627
L-Tyrosine	HMDB0000158	Simone et al.,2013
L-Alanine	HMDB0000161	Unconfirmed
L-Arginine	HMDB0000517	PMID:25700627
5-Hydroxyindole acetic acid	HMDB0000763	Unconfirmed
Creatine	HMDB0000562	PMID:28144886

The incidence of colorectal cancer (CRC) is second only to gastric cancer, esophageal cancer and primary liver cancer [31]. In recent years, the incidence of colorectal cancer in adolescents and young adults is higher. Endogenous metabolites have verified it have great potential in the early diagnosis and personalized treatment of CRC [32]. Using our method to predict metabolites with CRC, and sorting the score of results in descending order. 9 out of 10 metabolites are confirmed, which is described in Table 3.

**Table 3:** Top 10 metabolites of colorectal cancer identified by bi-random walks method.

Metabolite Names	HMDB ID	Evidences
1-Methyladenosine	HMDB0003331	PMID:7482520
N-Acetyl-D-glucosamine	HMDB0000215	PMID:27156840
Deoxyguanosine	HMDB0000085	PMID:27585556
Gentisic acid	HMDB0000152	PMID:25037050
N-Acetylgalactosamine	HMDB0000212	PMID:29507546
Saccharopine	HMDB0000279	Unconfirmed
L-Tyrosine	HMDB0000158	PMID:27275383
L-Glutamic acid	HMDB0000148	PMID:23940645
L-Histidine	HMDB0000177	PMID:20156336
Hypoxanthine	HMDB0000157	PMID: 28640361

Alzheimer's disease (AD), the most common cause of dementia, is a health problem that attracts increasing global attention and has a huge impact on human health [33]. Researchers developed various methods to identify AD related metabolites, for instance, using capillary electrophoresis-mass spectrometry to identify 9 metabolites are disease progression biomarkers [34]. Abnormal phospholipid metabolism is likely to lead to AD, and abnormal levels of metabolism is utilized to study AD by combining metabolomic-profiling approach [35]. 10 out of 10 predicted AD related metabolite were confirmed, as shown in Table 4.

**Table 4:** Identifying results of associated metabolites for Alzheimer's disease.

Metabolite Names	HMDB ID	Evidences
L-Tryptophan	HMDB0000929	PMID:17031479
L-Phenylalanine	HMDB0000159	PMID:23857558
Homocysteine	HMDB0000742	PMID: 29024723
L-Alanine	HMDB0000161	PMID:21292280
Uric acid	HMDB0000289	PMID: 30060474
Glycine	HMDB0000123	PMID:20858978
Homovanillic acid	HMDB0000118	PMID: 28166276
L-Tyrosine	HMDB0000158	PMID:24898638
Hypoxanthine	HMDB0000157	PMID:24898638
Cholesterol	HMDB0000067	PMID: 27773727

## Conclusions

There is increasing evidence that metabolites play an important role in the prediction, diagnosis and treatment of many complex diseases. In this paper, MDBIRW be used to predict the latent associations between metabolite and disease. The experimental results and case studies illustrate that the performance of MDBIRW is superior to that of other methods. The effective performance of MDBIRW mainly due to following factors. Firstly, the semantic disease similarity, metabolite functional similarity and Gaussian interaction profile kernel similarity were integrated. Secondly, by controlling the number of iterative steps in metabolite network and disease network, MDBIRW can make better use of the hierarchical information of the nodes in two subnetworks to achieve a higher prediction accuracy.

This method still has some limitations needing to be improved in future research. First, gaussian interaction profile kernel similarity of diseases and metabolites Overreliance on known metabolite-disease association, resulting in biased similarity calculations. For this problem, different data can be used to compute the similarities of diseases and metabolites such as GO data. In addition, we only used single data source, disease and metabolic data. Complex diseases are commonly caused by the interaction of multi-omics, thus, we will combine other omics data to improve prediction performance in the following study.

## Supporting information

S1 File The data file of metabolite-disease associations. (XLS)
